# Influence of body weight unloading on human gait characteristics: a systematic review

**DOI:** 10.1186/s12984-018-0380-0

**Published:** 2018-06-20

**Authors:** Salil Apte, Michiel Plooij, Heike Vallery

**Affiliations:** 1 Mechanical, Maritime and Materials Engineering (3mE), TU Delft, Mekelweg 2, Delft, 2628 CD Netherlands; 2 Motekforce Link, Hogehilweg 18–C, Amsterdam, 1101 CD Netherlands

**Keywords:** Body weight support, Rehabilitation, Gait characteristics

## Abstract

**Background:**

Body weight support (BWS) systems have shown promise as rehabilitation tools for neurologically impaired individuals. This paper reviews the experiment-based research on BWS systems with the aim: (1) To investigate the influence of body weight unloading (BWU) on gait characteristics; (2) To study whether the effects of BWS differ between treadmill and overground walking and (3) To investigate if modulated BWU influences gait characteristics less than unmodulated BWU.

**Method:**

A systematic literature search was conducted in the following search engines: Pubmed, Scopus, Web of Science and Google Scholar. Statistical analysis was used to quantify the effects of BWU on gait parameters.

**Results:**

54 studies of experiments with healthy and neurologically impaired individuals walking in a BWS system were included and 32 of these were used for the statistical analysis. Literature was classified using three distinctions: (1) treadmill or overground walking; (2) the type of subjects and (3) the nature of unloading force. Only 27% studies were based on neurologically impaired subjects; a low number considering that they are the primary user group for BWS systems. The studies included BWU from 5% to 100% and the 30% and 50% BWU conditions were the most widely studied. The number of participants varied from 1 to 28, with an average of 12. It was seen that due to the increase in BWU level, joint moments, muscle activity, energy cost of walking and ground reaction forces (GRF) showed higher reduction compared to gait spatio-temporal and joint kinematic parameters. The influence of BWU on kinematic and spatio-temporal gait parameters appeared to be limited up to 30% unloading. 5 gait characteristics presented different behavior in response to BWU for overground and treadmill walking. Remaining 21 gait characteristics showed similar behavior but different magnitude of change for overground and treadmill walking. Modulated unloading force generally led to less difference from the 0% condition than unmodulated unloading.

**Conclusion:**

This review has shown that BWU influences all gait characteristics, albeit with important differences between the kinematic, spatio-temporal and kinetic characteristics. BWU showed stronger influence on the kinetic characteristics of gait than on the spatio-temporal parameters and the kinematic characteristics. It was ascertained that treadmill and overground walking can alter the effects of BWU in a different manner. Our results indicate that task-specific gait training is likely to be achievable at a BWU level of 30% and below.

**Electronic supplementary material:**

The online version of this article (10.1186/s12984-018-0380-0) contains supplementary material, which is available to authorized users.

## Background

Body weight supported training (BWST) has shown promise in providing improvements in motor function, locomotion ability and balance in patients suffering from damage to the nervous system [1–6]. Example patient groups are spinal cord injury (SCI) patients, stroke patients and Parkinson’s disease patients. During BWST, a certain percentage of the patient’s body weight is supported by an overhead suspension system through a harness worn by the patient [7]. BWS systems enable physiotherapists to assess and correct gait patterns during interventions, without the obligation of providing full physical assistance [8]. In one of the earliest studies on this subject, Wernig et al. discovered that, with body weight supported treadmill training (BWSTT) for around 7 months, SCI patients having complete or partial paralysis could learn to perform voluntary bipedal stepping with joint stabilization and body weight bearing [9]. Patients with a paralyzed limb were able to walk short distances while bearing their own weight and in absence of joint stabilizers like knee braces. Recently, with the advent of robotic rehabilitation devices, the total duration of training and its precision can be increased even more without increasing the burden on the physiotherapists, thus enabling wider application of BWST [10, 11].

A BWS system is typically composed of an apparatus in which the patient is mechanically supported by a harness while walking on a treadmill or overground [8]. The constraints and support provided by the BWS system helps the subjects’ vertical alignment and stability of the trunk throughout ambulation [8, 12]. This, in turn, can provide neurologically impaired users the confidence to start rehabilitation early after surgery or trauma to regain balance and locomotion without the fear of a fall [8]. Furthermore, BWS systems also allow perturbation-based training of patients in a safe environment in order for the patients to improve their balance. BWS decreases lower-extremity load, thus facilitating step initiation [13]. When a treadmill is used, the treadmill can aid hip extension in the stance leg, critical to the initiation of swing phase, and supply temporal cues associated with stepping [14]. Although it is still debated [15], several studies indicate that task specificity in rehabilitation training is crucial [16]. BWST makes use of such task-oriented outlook with the aim of improving the performance of that task. Further benefits of BWST seem to be improved cardiovascular health, increased glucose tolerance and insulin sensitivity, growth in muscle mass, reduction in visceral fat and enhanced psychological well-being [17].

The theoretical underpinning of BWST as a clinical intervention is the concept of neuroplasticity [18]. The purpose of BWST is to supply the injured nervous system with necessary and appropriate sensory input signals for stimulating the intact spinal cord networks in order to facilitate their continued involvement even when supraspinal input is undermined [19]. Barbeau et al. first suggested the use of a treadmill and BWS for the gait rehabilitation of patients with SCI [20]. Since the study by Barbeau et al., other studies have reinforced the idea that BWST of persons with clinically complete or incomplete SCI induces functional re-organization of spinal neuronal networks, which leads to improvements in motor function and decreased muscle co-contractions [10, 18, 21–25].

It is still an open question how to choose a suitable level of body weight unloading (BWU) during locomotor training. Often, the selection is based on subjective judgement and visual inspection of the resulting gait pattern. It is known from research on motor control that even small adaptations of tasks may affect the corresponding movement strategy [26]. Therefore, choosing a level of BWU that preserves natural gait characteristics under BWU may improve the outcome of treatment [27, 28]. Therefore, the aim of this meta-analysis is to answer the central question:

‘How does body weight unloading affect gait characteristics?’

Thus, the primary goal of this paper is: (1) To quantify and analyze the influence of body weight unloading (BWU) on gait characteristics. In addition to this, two secondary goals are: (2.1) To study whether the effects of BWU differ between treadmill and overground walking; and (2.2) To investigate if modulated BWU influences gait characteristics less than unmodulated BWU. The scope of literature covered in this paper is limited to the research that is aimed towards using BWST for rehabilitation purposes and published between 1991 to 2016. It includes studies about walking under BWS for neurologically impaired adults and those with no known motor disorders. The pathologies included in this review are spinal cord injury, cerebrovascular accident (stroke) and Parkinson’s disease.

This paper is divided into three sections. The first section explains the methodology pursued while conducting the literature review. This is followed by a detailed description of the parameters used to study effects of BWU on gait and the results and trends for each of these parameters reported in existing experimental research. The paper concludes with a discussion on the important gait outcome measures studied in literature, the distinction between results for treadmill-based and overground studies and a overview of the experiments aimed at investigating effects of body weight unloading (BWU) on gait.

## Method

### Search strategy

Identification of potentially relevant literature was conducted through electronic search of four databases: Pubmed, Scopus, Web of Science and Google Scholar. The following search terms were utilized using the Boolean mode - (weight support OR weight unloading OR simulated gravity OR reduced gravity) AND (body OR gait OR locomotion OR characteristics OR rehabilitation OR overground OR treadmill OR spinal cord injury OR stroke OR parkinson’s OR walking). Searches were limited to studies based on adult human subjects performing a walking task, published in English language and up to the year 2016. These search results were extended by examining the references lists of returned articles. Apart from these searches, citations of the papers presenting the design of electromechanical body weight support systems were explored [29–34]. Literature about the effects of water immersion on human gait is not considered relevant due to the drag and damping produced by the viscosity of water [35, 36].

### Literature identification

The population of interest were both healthy individuals and individuals suffering from neurological disorders like SCI, stroke and Parkinson’s disease. Though the symptoms and effects of these disorders are different, they were combined into one group for the purpose of analysis. Since the number of studies for each disease was limited, we had to combine them for obtaining meaningful conclusions. While literature about the clinical outcomes of BWST in adults with other neuromuscular disorders is available [37, 38], studies about the influence of body weight unloading on gait biomechanics during training are missing. Though BWST is also utilized for pediatric rehabilitation [39–42], a combined meta-analysis of studies with adults and children as subjects would make it difficult to interpret the results. Consequently, the scope of this review is confined to experiments with adult participants. The relevant outcome measures are all gait characteristics including kinetic, kinematic and spatio-temporal parameters along with energy consumption, heart rate and muscle activity.

For an article to be included in this review, the source article had to describe: (1) whether an electromechanical BWS system was used; (2) nature of weight unloading; (3) treadmill/overground walking; (4) gait characteristics used and (5) a gait analysis experiment with at least one participant. The last criterion excludes any simulation-based studies. Despite inclusion of any particular study in this review, the data from that study was excluded from the meta-analysis if: (1) the experiment involved less than five participants (2) data of the clinical outcome of BWS training was presented instead of the data showing the influence of BWU on gait during body weight supported walking.

Gait data is excluded from the studies where effects of each BWU level are tested at different speeds and the studies in which only the change in gait parameters is mentioned [43–45]. Results of the experiments where assistive devices were used in combination with a BWS systems are not incorporated in the analysis [46–50]. Since provision of guidance through assistive devices can lead to a lower muscle activity and these effects can dominate over the influence of BWU [51], exclusion of the data from these studies improves the reliability of the statistical analysis. One paper presented data in the form of a linear regression instead of providing raw data [52]. As this might lead to misleading values of the coefficient of determination (R^2^), this data is also ruled out from the statistical analysis.

Results are also not included from the experiments featuring a BWS system with a tilted walkway [53–55], nearly-parabolic flight [56, 57], partial immersion in water [58], horizontal suspension systems [59], saddle-based body attachment [60–62] and air-pressure unloading force around lower body [63–65]. These different types of BWS systems might influence the gait differently than the more widely used harness-based vertical BWS systems [66, 67] and thus their exclusion from the selected literature.

### Data extraction

The following data was extracted from the selected literature: (1) BWS type; (2) treadmill or overground walking; (3) participants’ physiological condition (neurologically impaired or otherwise); (4) number of participants; (5) unloading conditions tested for and (6) gait characteristics investigated and their units of measurement (see Additional file 1). Mean values for each independent gait parameter were obtained from the studies.

### Study classification

The literature was classified based on three distinctions. First, treadmill and overground studies were distinguished. This nature of the walking environment is important, since it has been claimed to be a critical factor for facilitating the skill transfer to everyday movements [68]. For example, the walking speed chosen on a treadmill is typically not self-selected unlike overground gait [8]. In addition, a body-weight support system above a treadmill also provides relative assistance for propulsion, while the same does not necessarily hold for overground gait [69]. The training outcomes for treadmill-based and overground training might also be different. Field-fote et al. discovered that walking distance improved to a larger degree with overground training as compared to treadmill-based training for individuals with chronic motor incomplete SCI [70]. Second, studies of healthy subject (H) and those of subjects with neuromuscular impairments (referred hereafter as NI for brevity) were differentiated into two different groups. One could also distinguish between different patient groups, but due to the small amount of studies per patient group, it was decided to categorize all neurologically impaired subjects together. Finally, there is a distinction between constant and modulated BWU systems based on whether or not they are designed to modulate the unloading force.

**Fig. 1:**
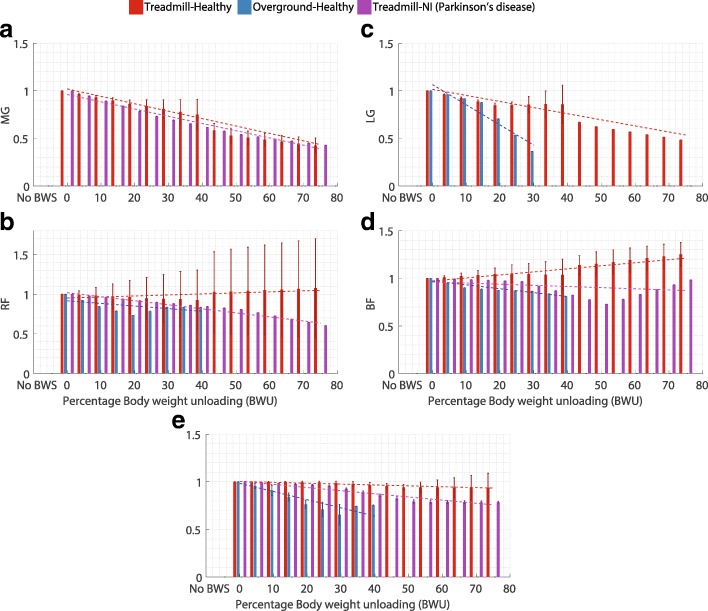
Flowchart for classification of studies into six categories which are indicated in colour. Similar colour scheme is followed in Figs. 3, 4, 5, and 6 in the results section

The subject results were classified into six categories (Fig. 1), based on the first two distinctions: (1) treadmill-healthy (TH); (2) overground-healthy (OH); (3) treadmill-NI (TN) with results for both legs considered together and (4) overground-NI with results for both legs considered together (ON). The last two categories were further divided into results for (5) non-paretic leg (TNN and ONN) and (6) the paretic leg (TNP and ONP). The outcomes for these groups will be shown throughout the results section.

The types of BWS systems were grouped into four different groups, based on the first and third distinction: (1) treadmill modulated (T-M); (2) overground modulated (O-M); (3) treadmill unmodulated (T-UM) and (4) overground unmodulated (O-UM). The difference in the outcomes between these groups is examined in the discussion section.

**Fig. 2:**
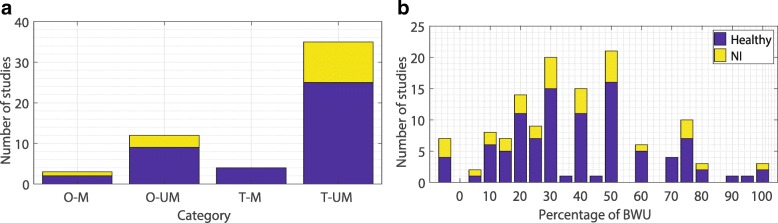
Summary of BWS studies where O:Overground, M:Modulated, T:Treadmill, UM:Unmodulated. Plot A shows the number of studies per category. Only 27% of these studies are based on subjects with neuromuscular disorders i.e the NI group. Plot B shows the number of studies for each level of BWU. The most investigated BWU level is 50%, followed by 30%, 40% and 20% respectively. NI represents the category of subjects with neuromuscular impairment

Figure 2 shows the number of studies found per category and the amount of BWU studied by them. Table 1 presents the studies as classified per type of BWS that they use.

**Table 1:** Classification of BWST literature based on nature of unloading force and walking environment. BWS studies based on individuals with neuromuscular impairment (NI group) are indicated in bold

	Treadmill-based	Overground
Modulated BWU	Franz 2007 [32], Franz 2008 [71], Van Thuc 2015 [124], Munawar 2016 [125] – *4 studies*	Morbi 2012 [130], Fenuta 2014 [49], **Fenuta 2014**^***a***^ [99] - 3 studies
Unmodulated BWU	Finch 1991 [43], Farley 1992 [61], Donelan 1997 [60], Kram 1997 [62], **Dietz 1997** [100], **Harkema 1997** [22], **Dietz 1998** [101], Colby 1999 [94], **Hesse 1999** [85], Stephens 1999 [78], Griffin 1999 [93], **Danielsson 2000** [95], Ferris 2001 [75], Ivanenko 2002 [79], Threlkeld 2003 [84], Grabowski 2005 [96], **Ferris 2004** [97], Van Hedel 2006 [80], **David 2006** [46], **Phadke 2007** [24], Thomas 2007 [81], McGowan 2008 [92], Aaslund 2008 [26], McGowan 2009 [76], Lewek 2010 [73], Klarner 2010 [47], Kuno 2012 [52], Goldberg 2013 [90], **Delussu 2014** [48], **Meyns 2014** [120], Van Kammen 2014 [83], Worthen-Chaudhari 2015 [45], Swinnen 2015 [50], Dragunas 2016 [12], Van Kammen 2016 [51] – *35 studies*	Patino 2007 [86], **Sousa 2009** [8], **Burgess 2010** [87], Wang 2011 [98], Serrao 2012 [74], Barela 2014 [91], Fischer 2015 [88], Fischer 2015 ^*a*^ [102], **Hurt 2015** [44], Fischer 2016 [109], Mun 2016 [89], Ye 2016 [122] – *12 studies*

^a^indicates two different publications by the same author/s in the same year. Studies by David et al. and Delussu et al. were conducted on GaitTrainer, a stepping plate based device [46, 48]

### Statistical analysis

The gait characteristics studied by the literature were categorized in gait kinematics, gait kinetics, metabolic parameters, and muscle activity, and are described in Table 2. Only studies investigating gait characteristics at different levels of BWU were considered for statistical analyses (see Table 3). For the literature comparing modulated and unmodulated BWU, only the results for the unmodulated condition were used for the statistical analysis [32, 71]. This enabled the comparison with other papers which use only unmodulated BWU. Apart from the gait characteristics presented in Table 2, other characteristics have been investigated in the literature concerning BWS systems, such as: (1) gait symmetry [8, 71]; (2) consistency of gait cycles [47]; (3) trunk movement [8, 26, 50]; (4) pelvic motion [50, 72]; (5) leg segment kinematics [8]; (6) joint power generation [32, 45, 73]; (7) nocioceptive flexion reflex [74]; (8) soleus H-reflex [24, 75]; (9) vertical impulse [76] and (10) horizontal trunk work [76]. These gait parameters were not analyzed either because there was only one study about them or in case of multiple studies, the available data was in a form that did not allow for comparison across literature.

**Table 2:** Categorization of gait characteristics

Group	Parameters
Kinematic parameters	1. Stride length 2. walking speed 3.cadence 4. single limb support phase 5. double limb support phase 6. total stance phase 7. hip angle range of motion (ROM) 8. knee angle ROM 9. ankle angle ROM
Kinetic parameters	10. Hip extension moment 11. hip flexion moment 12. knee extension moment 13. knee flexion moment 14. ankle joint moment 15. ankle joint impulse 16. antero-posterior ground reaction force (GRF) peak I 17. antero-posterior GRF peak II 18. vertical GRF peak I 19. vertical GRF peak II
Metabolic parameters	20. Energy cost of walking (ECW) 21. heart rate (HR)
Muscle activity	22. Medial gastrocnemius muscle (MG) 23. lateral gastrocnemius (LG) 24. rectus femoris (RF) 25. biceps femoris (BF) 26. tibialis anterior (TA)

**Table 3:** Classification of BWST literature based on nature of unloading force and walking environment, and studies considered for statistical analysis. BWS studies based on individuals with neuromuscular impairment (NI group) are indicated in bold

	Treadmill-based	Overground
Modulated BWU	Franz 2007, Franz 2008 – *2 studies*	Fenuta 2014 - 1 study
Constant BWU	Finch 1991, **Dietz 1997**, **Dietz 1998**, Colby 1999, **Hesse 1999**, Stephens 1999, Griffin 1999, **Danielsson 2000**, Ferris 2001, Ivanenko 2002, Threlkeld 2003, Grabowski 2004, Van Hedel 2006, Thomas 2007, McGowan 2008, Aaslund 2008, McGowan 2009, Lewek 2010, Goldberg 2013, Van Kammen 2014, Dragunas 2016 – *21 studies*	Patino 2007, **Sousa 2009**, **Burgess 2010**, Wang 2011, Barela 2014, Fischer 2015, Fischer 2015 ^*a*^, Mun 2016 – *8 studies*

^a^indicates two different publications by the same author/s in the same year

The investigated BWU levels were not uniform across the studies and were usually in increments of 10 to 20% BWU. Linear interpolation was used to obtain the values of gait parameter results at every 5% of unloading. This allowed comparison between studies at all percentages of BWU and bolstered the analysis by providing more data. However, no extrapolation was applied to extend the data beyond BWU levels available from the studies. For each study, individual parameters were normalized by taking a ratio with respect to their value at 0% BWU. This way the scaling process brought an uniformity in results and allowed comparison of trends across literature. By removing the dimensions attached to each parameter through scaling, comparison across different gait parameters was possible. For each of the four categories mentioned above, the mean and standard deviation (SD) for all gait parameters was calculated using the results of relevant studies.

Linear regression [77] was used to further analyze the response of the gait characteristics to the increase in % BWU. Linear regression was carried out separately for each of the six categories mentioned in Fig. 1. The slope (m) and the coefficient of determination (R^2^) for the gait parameters are presented in the results section. The slope ‘m’, which represents the change in the normalized parameter value per unit change in the % BWU, has units %^-1^. An ‘m’ value less than or equal to 1 x 10^-3^ %^-1^ was approximated as zero and the parameter was assumed to remain constant. They indicate that, in a given category, the % BWU was not a useful predictor for that gait parameter. A R^2^ value above 50% was considered as a good fit. For a given category (TH, OH, etc.), the R^2^ value was only calculated if the number of available raw data points was higher than 3. Since the data was normalized, for each category, the zero conditions for all the relevant studies were considered as one data point in total.

In case of the studies with treadmill, some of them [12, 52, 73, 78–83] investigate the gait characteristics at multiple walking speeds in addition to different BWU levels. In order to analyze their results together, the outcomes for a specific walking speed are selected. The experiment by Threlkeld et al. was conducted only at a single treadmill speed of 1.25 ms^-1^ [84]. In order to allow a reasonable comparison with the data from this study, data from other treadmill-based experiments with multiple speed conditions was chosen at the speeds as close to 1.25 ms^-1^ as possible (Table 4).

**Table 4:** Selected walking speeds for statistical analysis [12, 73, 78–83]

Study	Chosen walking speed
Stephens et al. 1999	0.9 - 1 ms^-1^
Ivanenko et al. 2002	1.1 ms^-1^
Van Hedel et al. 2006	1.5 ms^-1^ (2 ms^-1^ for joint angles)
Thomas et al. 2007	1.26 ms^-1^
Aaslund et al. 2008	1.2 ms^-1^
Lewek et al. 2010	1.2 ms^-1^
Van Kammen et al. 2014	1.8 ms^-1^
Dragunas et al. 2016	1.47 ms^-1^

## Results

**Fig. 3:**
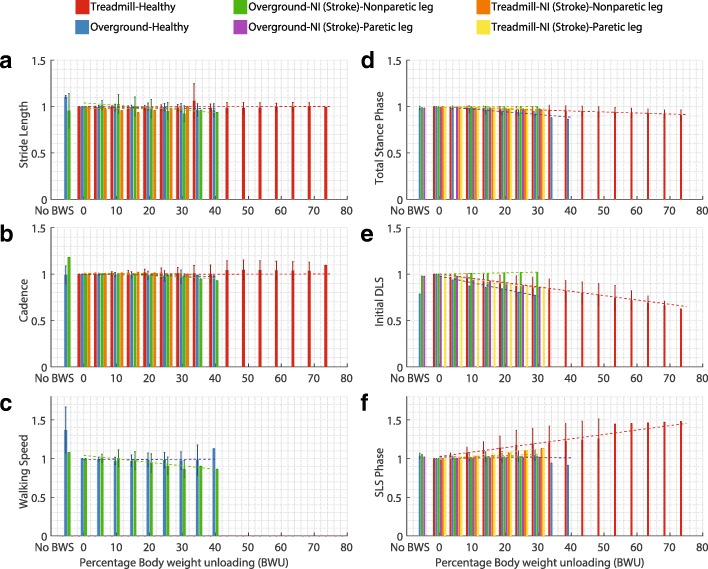
Influence of body weight unloading on gait spatio-temporal parameters where **a**. Stride length, **b**. Cadence, **c**. Walking speed, **d**. Total stance phase, **e**. Initial double limb support (DLS) phase, and **f**. Single limb support (SLS) phase. Vertical bars represent the normalized mean values, error bars depict standard deviation between studies and dashed lines illustrate the result of linear regression for each category. Absence of error bar at a BWU level indicates that the data was available from only one study

**Fig. 4:**
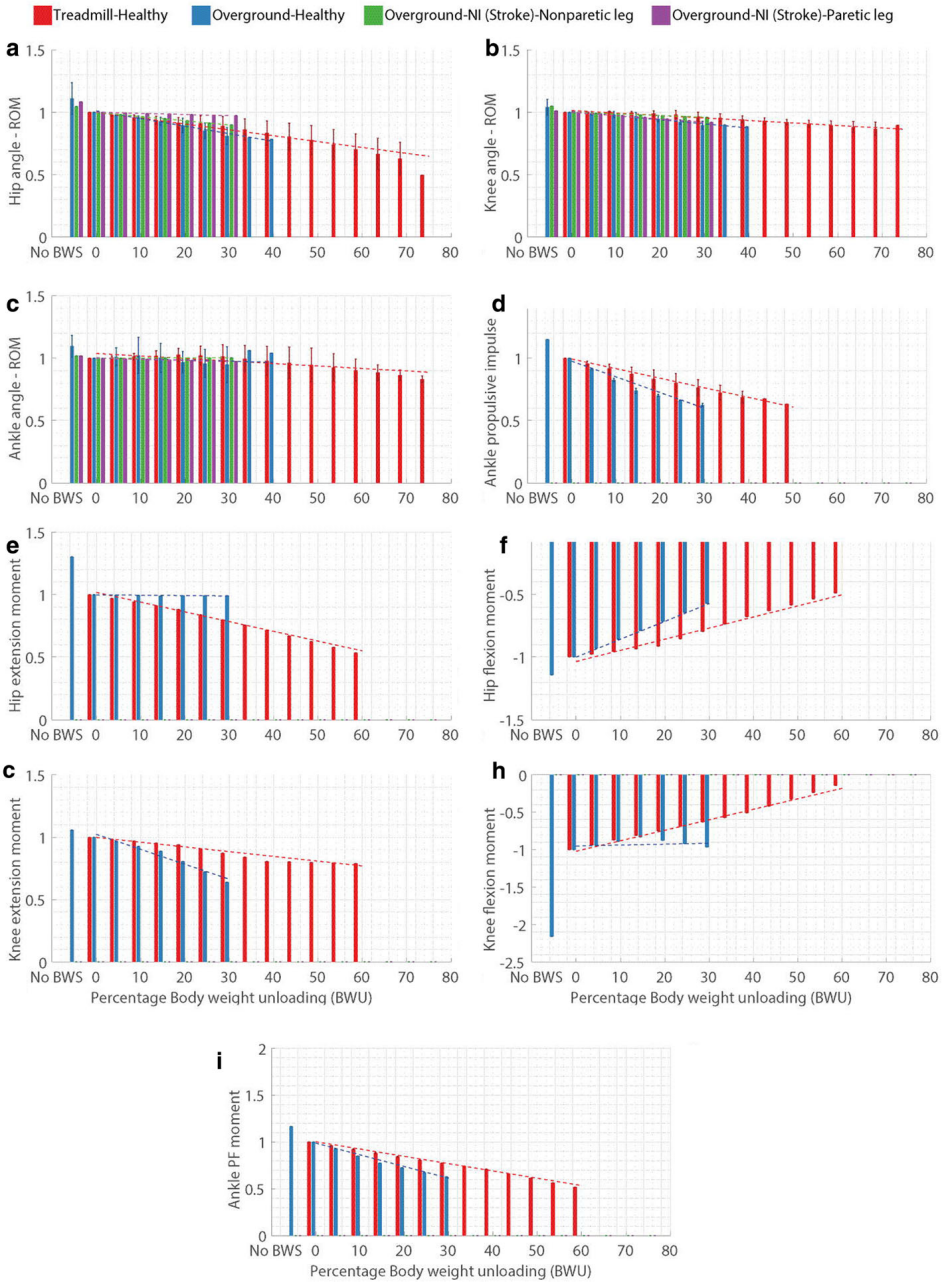
Influence of body weight unloading on joint kinematics and joint kinetics where **a**. Hip joint angle range of motion (ROM), **b**. Knee joint angle ROM, **c**. Ankle joint angle ROM, **d**. Ankle propulsive impulse, **e**. Hip extension moment, **f**. Hip flexion moment, **g**. Knee extension moment, **h**. Knee extension moment, and **i**. Anke plantarflexion moment. Extension and flexion moments are represented by positive and negative signs to imply opposite directions. Vertical bars represent the normalized mean values, error bars depict standard deviation between studies and dashed lines illustrate the result of linear regression for each category. Absence of error bar at a BWU level indicates that the data was available from only one study

**Fig. 5:**
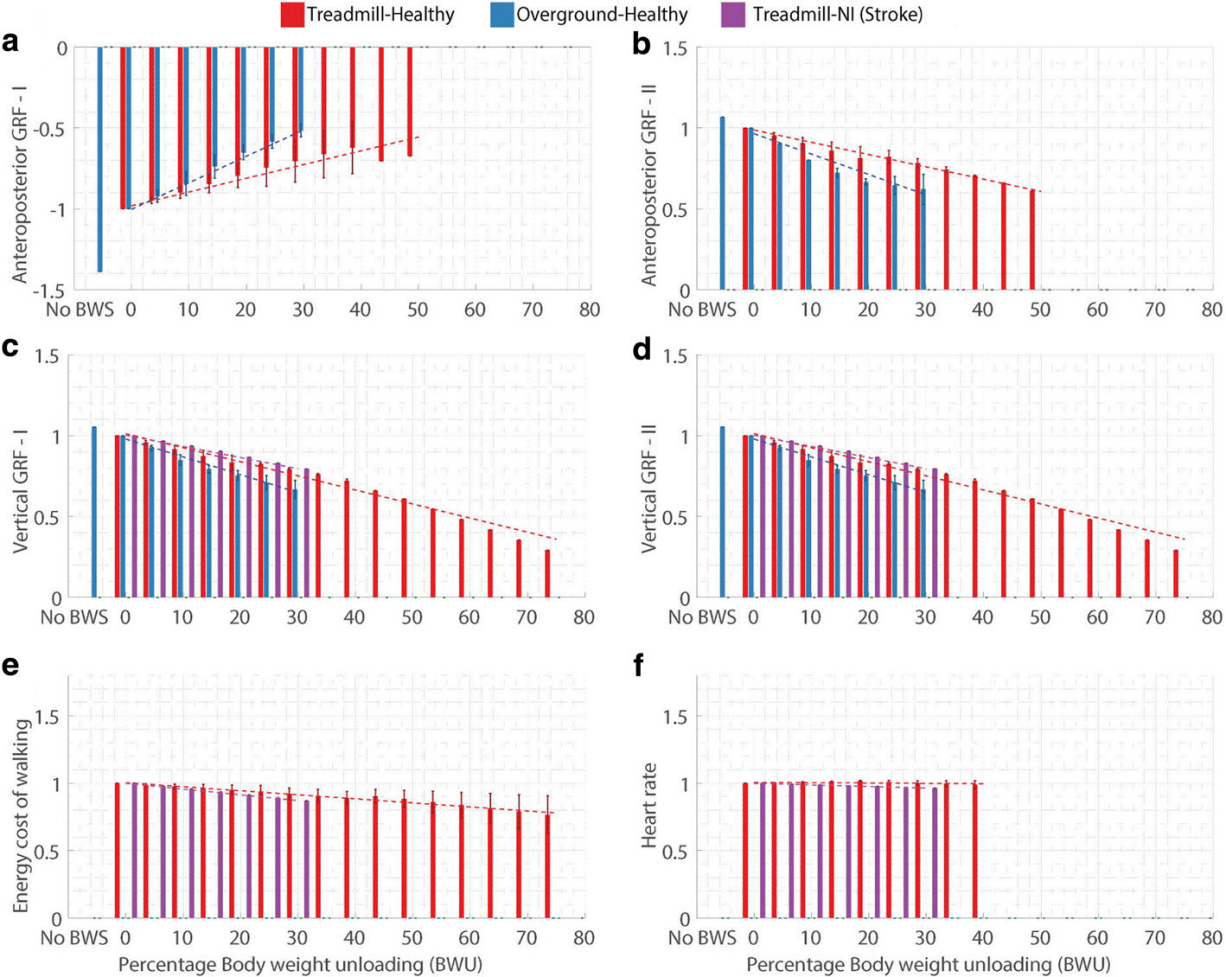
Influence of body weight unloading on ground reaction forces (GRF) and metabolic parameters where **a**. Anteroposterior GRF negative (deceleration) peak, **b**. Anteroposterior GRF positive (acceleration) peak, **c**. Vertical GRF peak I, **d**. Vertical GRF peak II, **e**. Energy cost of walking, and **f**. Heart rate. Vertical bars represent the normalized mean values, error bars depict standard deviation between studies and dashed lines illustrate the result of linear regression for each category. Absence of error bar at a BWU level indicates that the data was available from only one study

**Fig. 6:**
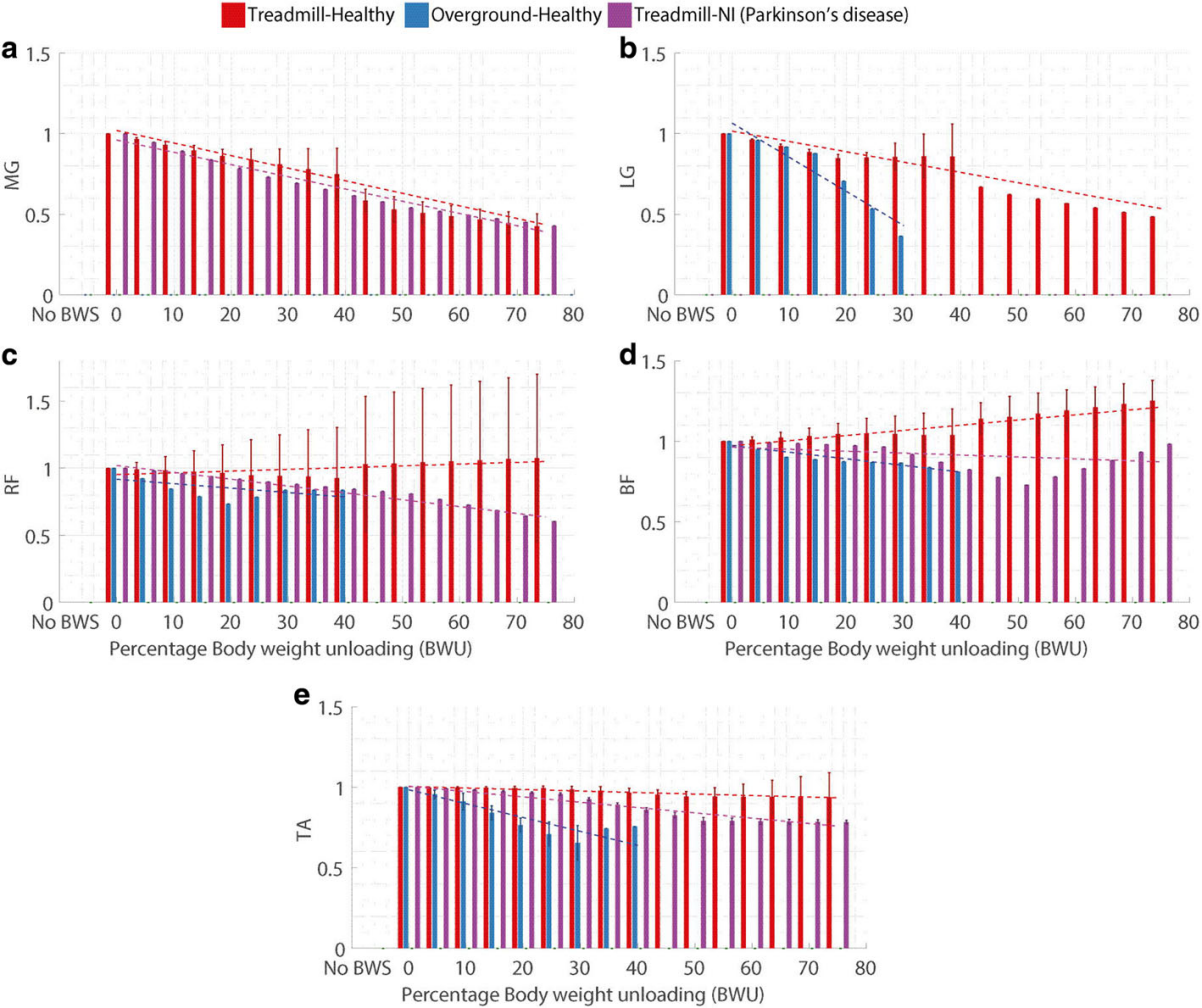
Influence of body weight unloading on mean muscle activity over gait cycle where **a**. Medial gastrocnemius (MG), **b**. Lateral gastrocnemius (LG), **c**. Rectus femoris (RF), **d**. Biceps femoris (BF) long head and **e**. Tibialis anterior (TA). Vertical bars represent the normalized mean values, error bars depict standard deviation between studies and dashed lines illustrate the result of linear regression for each category. Absence of error bar at a BWU level indicates that the data was available from only one study

For every gait parameter (Table 2) and every category (Fig. 1), the normalized values at the available % BWU levels were plotted in Figs. 3, 4, 5 and 6. The aim of these plots is to understand if the parameters follow a specific pattern with respect to the % BWU. The results are organized according to the different categories of gait parameters (Table 2).

### Gait kinematics

The values (Fig. 3a) of stride length (SL) were reported by 12 studies [8, 12, 32, 73, 80, 81, 84–89]. None of four groups showed a specific behavior for the magnitude of stride length. In case of the experiment by Franz et al., SL changed by -3% for the unmodulated 20% BWU as compared to -1% for the 20% modulated BWU [32]. 10 papers described the influence of % BWU on cadence (Fig. 3b) [8, 26, 32, 80, 81, 84–88]. ON group presented a decrease in cadence while other three groups did not present any definite pattern. Modulated BWU at 20% support led to a -0.78% difference in cadence compared to -3.2% for unmodulated BWU [32].

Data for walking speed (Fig. 3c) was extracted from 5 studies [8, 86–89]. The OH group showed a considerable decrease in gait speed from walking without harness to 0% BWU but no specific behavior beyond 0% BWU. In case of ONN group, gait speed decreased for BWU greater than 0% but there was no agreement within the studies for the slope (m) of the decrease. Results from the experiments involving treadmill were not presented since the participants usually walk at a predetermined speed on the treadmill.

Results for the proportion (percentage) of total stance phase (ST) (Fig. 3d), initial double limb support phase (iDLS) (Fig. 3e) and single-limb support phase (SLS) (Fig. 3f) in the gait cycle were taken from 10 studies [8, 78–80, 83–86, 88, 89]. The proportion of swing phase (SW) and terminal double limb support phase (tDLS) can be inferred from the above presented values. ST remained almost constant for all groups except TNN and OH, where it decreased. ST also decreased for the TH group but there was no agreement within the studies for the slope (m) of the decrease. iDLS stayed constant for the ONN category but reduced in case of all other groups. SLS did not show a specific pattern for the OH group while it remained unchanged for the ONP group. However, SLS increased for other four groups.

It is important to note that data for gait phases for the ONN and ONP groups was obtained from a single research paper [8]. Furthermore, results for all the spatio-temporal parameters for the TN category were also available from only study [85].

The ROM (range of motion) data of all three leg joints for the overground-neuromuscular impairment group (ONN and ONP) was obtained from a single study [8]. The R^2^ values for these two groups are 100% as this study contained data for only two conditions, 0 and 30% BWU [8]. Data for hip joint ROM (Fig. 4a) was analyzed from 7 studies [8, 32, 80, 84, 86, 88, 89]. Hip ROM decreased for TN, OH and ONN groups but remained roughly unchanged for the ONP group. However, in case of the OH group, the ROM reduced considerably after 30% BWU. The change in hip ROM for modulated and unmodulated 20% BWU was -1.21% and -11.41% respectively [32].

6 studies were used to obtain the data on knee joint ROM (Fig. 4b) [8, 80, 84, 86, 88, 89]. Rise in % BWU led to a reduction in knee joint ROM for all four groups. Ankle joint ROM (Fig. 4c) results were extracted from 8 studies [8, 32, 75, 80, 84, 86, 88, 89]. Ankle ROM almost remained constant for the neurologically impaired participant groups i.e. ONN and ONP. Contrary to this, it did not show any specific behavior for the healthy groups i.e. TH and OH. In case of modulated BWU, modulating led to 5.86% change in ankle ROM as compared to the 5.21% for unmodulated unloading [32].

### Gait kinetics

The data for hip and knee moments was obtained from 2 studies [88, 90], for ankle plantarflexion (PF) moment from 3 studies [73, 88, 90] and ankle propulsive impulse from 4 studies [73, 88, 91, 92]. Except for ankle propulsive impulse, data for the OH [88] and TH [90] groups for all other parameters was obtained only from one study each. In Fig. 4, flexion and extension moments are presented with negative and positive signs respectively to indicate opposite directions.

Ankle impulse and ankle PF moment decreased for both the TH and the OH groups. Hip extension moment and knee flexion moment remained roughly constant for the OH group (up to 30% BWU) while they reduced for the TH group. However, hip flexion and knee extension moments reduced for both the groups.

Data for the anteroposterior (AP) ground reaction force (GRF) was obtained from 5 papers [32, 73, 86, 91, 92] and for the vertical GRF from 6 papers [32, 73, 79, 85, 86, 91]. However, it should be noted that the data for the GRF for the TN group was from a single research study [85].

The peak values of AP GRF (AP GRF I - negative and AP GRF II - positive peaks) and vertical GRF (first and second peak) were considered for the statistical analysis. AP GRF values decreased for both TH and OH categories while vertical GRF reduced in magnitude for the TH, TN and OH categories. The reduction was consistently larger for the OH group as compared to the other two groups.

For the AP GRF I and 1^st^ vertical GRF peaks, the results for 20% modulated unloading were closer to 0% BWU for TH group than the 20% unmodulated unloading [32, 32]. However, for the AP GRF II and 2^nd^ vertical GRF peaks, it was vice-versa [32, 71].

### Metabolic parameters

Outcomes for energy cost of walking (ECW) were acquired from 5 studies and reported in terms of the *VO*_2_ max (volume of maximal oxygen uptake) [81, 93–96]. ECW (Fig. 5e) showed a similar decreasing trend for both the TH and TN groups. Data for heart rate was obtained from 3 papers [81, 94, 95]. Heart rate (Fig. 5f) did not show any specific trend for the TH category while it showed slight reduction for the TN category.

### Muscle activity

Muscle activity was considered in terms of the magnitude of the EMG signal as an average value over the entire gait cycle. Five muscles were examined: (1) medial gastrocnemius (MG); (2) lateral gastrocnemius (LG); (3) tibialis anterior (TA); (4) rectus femoris (RF) and (5) biceps femoris long head (BF). Apart from the studies considered for statistical analysis (mentioned below), other studies also investigated the influence of BWU on muscle activity [43, 78, 80, 83, 85, 86, 92, 97–99]. However, the relevant data for the average value of EMG signal from these papers was not available and hence they were excluded.

#### Extensor muscles

MG muscle (Fig. 6a) showed a decrease in muscle activity with the increase in % BWU for both the TH [73, 75, 94, 100, 101] and the TN categories [100, 101]. LG muscle (Fig. 6b) presented a reduction in magnitude for both the TH [73, 79] and the OH groups [102]. For the RF muscle (knee extensor, Fig. 6c), two groups, TH [79, 94, 100] and OH [89] did not show a any clear trend while the TN group [100] presented a decrease in the magnitude of muscle activity.

#### Flexor muscles

In case of the BF muscle (Fig. 6d), the TN group [100] and TH group [79, 94, 100] failed to show any consistent pattern in muscle activity while the OH group showed a clear decrease [89]. TA muscle (Fig. 6e) activity reduced for the TN [100, 101] and OH [89, 102] groups but did not present a consistent behaviour for the TH group [75, 79, 100, 101].

### Summary

The above presented results are summarized in Table 5. Since the statistical analysis covered only the unmodulated BWU studies, results from the modulated BWU were shown separately in this section. Modulated BWU at 20% showed lower deviation as compared to 20% unmodulated BWU for stride length, hip angle ROM, anteroposterior GRF peak I and vertical GRF peak I. For ankle angle ROM, the values were comparable while anteroposterior GRF peak II and vertical GRF peak II showed higher deviation for modulated BWU.

**Table 5:** Summary of the influence of BWU level on gait characteristics, where italic*: no definitive trend across studies, bold: gait parameter remains almost constant, —: no studies, NA: *R*^2^ not calculated since the number of available data points was less than four. Number of studies (*n*), slope (*m* %^-1^) and % *R*^2^ values for the linear regression of each gait parameter are written respectively. In cases where the magnitude of gait parameters is measured separately for non-paretic (T/OTN) and paretic legs (T/OTP), slope for the non-paretic leg is mentioned first. E: extension, F: flexion, PF: plantarflexion, ECW: energy cost of walking, MG: medial gastrocnemius, LG: lateral gastrocnemius, TA: tibialis anterior, RF: rectus femoris and BF: biceps femoris long head

Gait Characteristics	Treadmill						Overground					
	Healthy			NI			Healthy			NI		
	n	m	R^2^	n	m	R^2^	n	m	R^2^	n	m	R^2^
		×10^−3^	%		×10^−3^	%		×10^−3^	%		×10^−3^	%
Kinematic parameters												
1. Stride length	5	*-0.03* ^*^	0	1	*0* ^*^	0	4	*-0.5* ^*^	4.1	2	-2.6^*^	29.7
2. Cadence	5	*0.1* ^*^	0.1	1	*0* ^*^	0	3	*-0.7* ^*^	5.4	2	-1.5	65.1
3. Walking speed		—			—		4	*0.1* ^*^	0	2	*-4.5* ^*^	48.5
4. Gait phases - ST	4	*-1.1* ^*^	41.7	1	-1.4; **-0.9**	NA	3	-2.9	71.3	1	**0.1**; **-0.8**	NA
5. Gait phases - iDLS	3	-4.7	74.8	1	—; -4.7	NA	1	-7.2	93.6	1	**0.7**; -4.9	NA
6. Gait phases - SLS	2	5.7	61.9	1	1.9; 4.4	NA	3	*-0.3* ^*^	0.5	1	1.1; **0.6**	NA
7. Hip joint ROM	3	-4.7	76.6		—		3	-6	80.3	1	-3.4; **-0.9**	NA
8. Knee joint ROM	2	-2	80.8		—		3	-3.3	79.3	1	-1.3; -2.7	NA
9. Ankle joint ROM	4	*-2* ^*^	37.8		—		3	*-0.9* ^*^	1.4	1	**0.1**; **-0.9**	NA
Kinetic parameters												
10. Ankle impulse	2	-7.7	93.9		—		2	-12.6	94.9		—	
11. Hip E moment	1	-7.8	99.2		—		1	**-0.3**	NA		—	
12. Hip F moment	1	-8.9	97.1		—		1	-14	NA		—	
13. Knee E moment	1	-3.8	92.8		—		1	-12	NA		—	
14. Knee F moment	1	-14	98.9		—		1	**-1**	NA		—	
15. Ankle PF moment	2	-7.8	99.4		—		1	-12	NA		—	
16. AP GRF peak - I	3	-8.5	80.2		—		2	-16.4	96.3		—	
17. AP GRF peak - II	3	-7.6	91.6		—		2	-12.6	87.7		—	
18. Vertical GRF - I	3	-8.3	95	1	-6.6	NA	2	-9.6	99.1		—	
19. Vertical GRF - II	3	-8.7	96	1	-6.9	NA	2	-11	93.8		—	
Metabolic parameters												
20. ECW	5	-3	70.2	1	-4.3	NA		—			—	
21. Heart rate	2	*-0.3* ^*^	6.6	1	-1.2	NA		—			—	
Muscle activity												
22. EMG - MG	5	-7.8	83.1	2	-7.6	96.7		—			—	
23. EMG - LG	2	-6.4	72.1		—		1	-21.2	NA		—	
24. EMG - RF	3	*1.3* ^*^	1.3	1	-5.1	95.9	1	*-3.3* ^*^	30.5		—	
25. EMG - BF	3	*-3.2* ^*^	42.5	1	*-1.2* ^*^	8.4	1	-4.1	88.6		—	
26. EMG - TA	4	*-0.9* ^*^	12.4	2	-3.3	88	2	-8.76	73.3		—	

## Discussion

This paper combined all studies on the effect of BWU on the gait in order to analyze how body weight unloading influences gait characteristics. In this section, we address: (1) the general trends in how BWU influences different gait parameters; in addition, we address the two sub-goals of our paper, (2) the differences between the influence of BWU in treadmill and overground walking environments (3) a comparison between modulated and unmodulated BWS; and provide (4) an overview of the literature on BWS studies.

### Influence of BWU on gait characteristics

The trends for each category of gait parameters (Table 2) are discussed here, followed by a discussion on the task specificity of walking under BWU. These categories of gait parameters correspond to the categories used to structure the ’Results’ section. To put the results into perspective, we also present a comparison of our results with existing research on human gait in low-gravity environments.

**i. Gait kinematics:** Stride length did not present a consistent behaviour for all relevant groups (Table 5). Cadence showed a decreasing trend for ON group but an inconsistent trend for TH, TN and OH groups. Total stance phase presented inconsistent behaviour for only TH group, Ankle joint ROM for both TH and OH groups, and walking speed for OH and ON categories (Table 5). The gait spatio-temporal parameters like cadence and gait phase proportions, and the kinematic parameters like ankle and knee ROM show a weak influence of unloading force up to 30% BWU. However, 13 studies (9 out of 16 for overground walking) investigated the effects of %BWU only up to 30%. For gait characteristics and participant groups where the R^2^ values lies between 50% and 60%, there is usually a similar trend (downward/upward) for all considered studies but little consistency in the slope (m) values across these studies.

**ii. Gait kinetics:** In case of the healthy groups, the relative magnitude of change in joint kinetics and ground reaction forces (GRF) is higher than that in joint kinematics and spatio-temporal parameters (Table 5). In addition to larger absolute values of the slope (m), gait kinetic parameters also generally show higher *R*^2^ values than gait kinematics, thus indicating a stronger agreement between different studies. For the TH group, gait characteristics involved in the push-off phase, like ankle plantarflexion moment, knee flexion moment, and ankle propulsive impulse, show a strong influence of BWU. As expected, BWU also has a notable influence on the magnitude of GRF peaks since the unloading force directly supports the user’s weight thereby reducing reaction forces from the ground.

**iii. Metabolic parameters:** Table 5 shows that the energy cost of walking decreases with the increase in BWU level for the TH group while heart rate remains roughly constant. Studies by Richter et al. and Harvill et al. report a similar trend. [67, 103]. An earlier review by Wortz et al. also states that at lunar gravity (similar to around 83% BWU), human locomotion entails significantly lower energy cost than at terrestrial gravity conditions (similar to 0% BWU) [104]. However, this reduction in energy requirement is not limited to a walking gait. In fact, as the BWU level is raised or the effective gravity lowered, the energy cost for a running or skipping gait decreases more rapidly than the cost for walking gait [61, 105]. Thus, at high BWU levels, walking is not the cheapest mode of locomotion in terms of energy cost. It is hypothesized that leg movement and thus the mode of locomotion is modulated to minimize the energy consumption during locomotion [106, 107]. This might lead to changes in gait at high levels of unloading which would be difficult to detect due to the smooth transitions [62, 108], thus adversely affecting the task specificity of BWS training.

**iv. Muscle activity:** With the increase in BWU level, muscle activity typically showed a higher reduction in magnitude than the kinematic parameters (Table 5). The gastrocnemius muscles (lateral & medial) presented a stronger influence of BWU as compared to other muscles. Gastrocnemius is involved in ankle plantarflexion (PF) and the large reduction in muscle activity due to BWU corresponds correctly with the large reduction in the ankle PF moment, as seen in the Table 5. However, other muscles did not show a consistent behaviour for some groups, like TA muscle for the TH group, RF muscle for both TH and OH groups, and BF muscle activity for TH and TN groups (Table 5).

**v. Summary:** The optimum amount of BWU is an important factor for gait rehabilitation training and consequently a key topic of study on the effects of BWU on gait [23]. From the results of this paper, it can be seen that the increase in the amount of BWU influenced all the 26 gait parameters listed in Table 5. While the percentage of single limb stance (SLS) phase increased with the increase in BWU, almost all other parameters showed a decreasing trend.

#### Task-specificity of gait under BWU

Curvature patterns of the joint trajectories remain roughly similar despite of the increase in BWU level up to 30% [8, 32, 47, 47, 80, 84, 86, 88, 89]. It is possible that the changes in the hip and knee adduction moment and ankle propulsive impulse and the changes in COP trajectory allow the kinematic patterns to remain similar [79, 109]. Thus, it can be inferred that up to 30% BWU force can be applied without significantly modifying the kinematic and spatio-temporal parameters associated with gait, which may be beneficial for the outcome of treatment [28]. This result from the meta-analysis aligns well with what other researchers already suspected in their separate studies [43, 45, 70, 80, 84, 88]. Of course, in some cases a higher amount of BWU might be necessary, for instance when patients find it difficult to bear their weight even with 30% BWU.

#### Comparison with literature on reduced gravity

Besides rehabilitation, BWS systems have been used to study the effects of reduced gravity on gait for the purpose of space exploration [35, 53, 54, 56, 59, 110–112]. The study by Richter et al. reviewed this literature and a comparison of results with that review is presented here. A separate comparison is also provided with the results by Harvill et al. for locomotion at lunar gravity [103], since these were not covered in the review by Richter et al.

Harvill et al. studied the effects of reduced gravity on gait for the purpose of space exploration while the paper by Richter et al. reviewed other literature on this topic. Regarding gait spatio-temporal parameters, both the papers reported a decrease in stance phase duration, a corresponding increase in swing phase duration but no specific trend for stride length and cadence. Richter et al. noted a higher dependence on walking speed for both stride length and cadence. These results are in agreement with our findings (Table 5).

In case of joint kinematics, both of these papers described a reduction in hip ROM and knee ROM. Harvill et al. noted a decrease in ankle ROM contrary to the inconsistent behavior reported by Richter et al. However, Richter et al. noted a very high effect size for hip and knee ROM unlike our results which show a weaker influence (Table 5). A possible explanation for this difference is that Richter et al. only analyzed gait parameters at very high (>60%) BWU levels.

According to Richter et al., joint impulses, energy cost of walking and heart rate showed higher reduction compared to kinematic parameters due to the decrease in gravity. GRF presented the highest influence of gravity in both the studies. In addition to showing that joint moments also show a large influence of simulated gravity (BWU level), our findings corroborate these results. The only exception is heart rate, for which we did not find any consistent behavior. Joint moments, impulses and GRF directly reflect the oscillation of the COM during gait, so their changed behavior under BWU shows that gravity plays an important role in COM oscillation.

### On treadmill vs overground studies

Comparison of results for the gait in overground (OG) and treadmill (TM) studies shows small but important differences (Tables 5 and 6) in gait characteristics. The OH group presents a greater influence of BWU on all gait parameters except single limb stance phase, hip extension moment and knee flexion moment. The TH group shows greater influence for these three parameters. Only in case of gait phases, neurologically impaired individuals show relatively similar results for both the walking conditions. This is in agreement with the conclusions from existing literature on walking without body weight support. If the treadmill speed is not set to match the preferred overground walking speed, differences arise between treadmill and overground walking [113–117]. These differences are prominent if the participants walk at self-selected walking speed on the treadmill which is not equal to the preferred speed in overground walking [118]. Thus, if the participants are not able to attain the preferred overground speed on a treadmill, the training might lose its task-specific nature [119].

**Table 6:** Summary of data in Table 5 – Trends for gait parameters which show different behavior in TM and OG environments

Affected parameter	Group	Treadmill	Overground
1. Cadence	Patients	Inconsistent	Decreasing
2. Stance phase %	Healthy	Inconsistent	Decreasing
3. SLS phase %	Healthy	Increasing	Inconsistent
4. BF muscle activity	Healthy	Inconsistent	Decreasing
5. TA muscle activity	Healthy	Inconsistent	Decreasing

Walking on a treadmill shows that both the treadmill speed and the amount of unloading have considerable influence on gait parameters [73, 80, 83, 90, 91, 120–122]. Cadence and stride length are affected more by the treadmill speed than by the percentage of BWU, except for above 75% BWU [67, 80]. The relative duration of gait cycle phases and consequently the joint angle patterns and the muscle activation patterns are influenced by the treadmill speed. Joint torques, ankle power generation, GRF profiles and pelvic excursions are also affected.

Treadmill walking may lead to confounding effects of BWU on gait characteristics and reduce the effectiveness of BWST [109]. While the data from the overground experiments shows that the walking speed changes beyond 10% of unloading, treadmill forces the participants to walk at a constant speed, which can result in unnatural gait dynamics. However, modulation of treadmill speed according to the amount of unloading provided might help to retain the natural gait pattern.

In case of the OH group, there was a reduction in gait speed from unsupported locomotion to walking in a harness at 0% BWU. A reasonable explanation for this observation is the requirement from users to pull the BWS system along while walking. Though overground walking seems more suited to gait training, pulling the BWS system forward against resistance, for example caused by friction, can make it difficult for the users to maintain a comfortable walking speed [8, 86]. However, using a motor-actuated winch system to pull the BWS system may help to ameliorate this problem [88].

### On modulated vs unmodulated support.

In the method section, we made a distinction between modulated and unmodulated support. Although there has been little research into modulated support, this section discusses the potential benefits of such systems as found in literature.

A BWS system should account for an individual’s specific physiological limitations and promote efficient locomotion patterns in order to provide optimal rehabilitation [123]. It has been claimed that modulation of unloading force can enable appropriate ground contact and limb motion while allowing gait spatio-temporal parameters like walking speed, cadence and stride length to be comparable to the values during unsupported walking [32]. Franz et al. designed a BWS system which controlled the unloading force based on gait cycle phases and conducted an experiment to compare it against a BWS system with constant unloading force [32, 71]. They compared the difference in the values of stride length, cadence, hip and ankle joint ROM, ankle power generation and GRF for constant and modulated 20% BWU conditions. The movement patterns and the magnitude of these parameters, except for anteroposterior GRF (deceleration) and 2^nd^ peak of vertical GRF, were closer to unsupported walking in case of modulated BWU.

Van Thuc et al. followed another approach towards modulation of unloading force; controlling the direction and magnitude of force according to the center of pressure (COP) trajectory [124]. They observed that the COP trajectory produced as a result of modulated BWU resembled that of unsupported walking more closely, as compared to the one with constant BWU.

Munawar et al. controlled the unloading force with the aim of offsetting the inertial forces of the user’s body dynamically [125]. They reported the pattern of vertical GRF for modulated BWU to be similar to that of unsupported walking. Ivanenko et al. and Fenuta et al. also conducted experiments with a modulated BWS system but did not report any comparative results between modulated BWU and constant BWU [79, 99]. Thus, it can be concluded prima facie that modulated unloading force generally led to less difference from the 0% BWU condition than unmodulated unloading.

### On body weight support studies

This paper compared 54 studies in the terms of the effects of the BWU on gait, published from the year 1991 to 2016 (refer to Additional file 1). Of these 55 studies, 30 are from the period 1991–2010 (20 years) while the remaining 24 are published 2010 onwards (6 years). This shows an increasing interest in the potential of BWST as a neurological rehabilitation tool.

Only 27% of the studies are based on individuals with either one of the neurological disorders (Stroke, Spinal cord injury and Parkinson’s disease) as participants (Fig. 2). This proportion is low considering that neurologically impaired individuals are the primary user group for BWS systems in general and rehabilitation tools in particular. The number of participants for the studies ranges from 1 to 28, with an average of 12 participants. In addition to this, only 53% of these publications explicitly state the use of randomization in the experimental protocol. This is in stark contrast to clinical studies, which generally include higher number of participants and are randomized by design [126–128]. Clinical studies were not included in this review since we could not find clinical trials which also presented gait parameter data during BWS training along with the post-training data. Secondary outcomes presented by clinical studies are also generally only assessed after training and so without BWS. The review by Richter et al. reported a similarly low methodological quality of studies investigating the influence of low gravity on human gait [67]. The low number of participants and the lack of randomized trials can both lead to suboptimal study design [129]. The proportion of studies investigating modulated BWS systems is around 13%, with the average number of participants being 6. These low numbers indicate a strong necessity for further research on BWS systems providing modulated unloading force.

The amount of BWU used for experiments ranges from 5 to 100%, with almost all studies utilizing different combinations and magnitudes of BWU (Fig. 2). Apart from the amount of unloading, the gait characteristics tested also vary substantially from one study to another. This suggests that there is no agreement within the research community over the appropriate levels of BWU for testing and the relative importance of gait parameters to be examined.

### Limitations of this review

The limitations of this study are presented and discussed below. First and foremost limitation is that the results from different patients groups (SCI, stroke and Parkinson’s disease) are pooled together and analyzed as a whole. This was done due to the paucity of studies based on subjects with a neuromuscular disorder. Pooling results together provided a large enough sample size for a meaningful statistical analysis. In order to minimize the distortions in the results due to different pathologies, data from each paper was normalized with respect to the value at 0% BWU. This normalization process shifted the focus of the analysis from absolute values of gait parameters to the trends followed by these parameters. However, upon closer inspection, it can be seen that the data for three neuromuscular disorders is for different gait parameters and no gait parameter has combined data from more than one of these disorders. Muscle activity data included in the statistical analysis is limited to subjects with Parkinson’s disease [100, 101] and while data for all other gait parameters is limited to stroke patients [8, 44, 85, 87, 95]. The SCI group [24] is not included in the final statistical analysis. Thus, the limitation of pooling different patient groups together in the analysis did not actually lead to inaccuracies in the results.

The second limitation is the combined analysis of experimental results based on different BWS systems. There are not enough studies for each BWS system to analyze the results separately. The third limitation is that experiments differing in usage of arms were also pooled together due to the limited number of studies. However, to improve consistency of data, only vertical BWS systems based on a harness-based attachment system were included in the analysis. This decision was taken based on the assumption that evaluating one only type of BWS system will reduce the artifacts introduced in the results by the BWS system.

Finally, this review is limited by the lack of a single metric to characterize and compare human gait. Furthermore, it is difficult to rank gait characteristics based on their importance to gait. Depending on the context, a small change in one gait parameter might be more important than a larger change in another. As a result of this, gait parameters were selected based on their frequency of use in practice, and a large number of gait parameters (26 in total) were analyzed, despite the scarcity of relevant studies for some of these parameters.

## Conclusion

This paper studied the influence of body weight unloading (BWU) on gait parameters through a meta-analysis. The results were grouped based on the physiological condition of the subjects (healthy or neurologically impaired), the type of walking environment (treadmill or overground) and the nature of unloading force (modulated or unmodulated). For healthy subjects, BWU influenced all gait characteristics except stride length, cadence, walking speed, and heart rate, where the influence was minimal. BWU affected all gait characteristics in case of the NI (neuromuscular impairment) group, but the number of characteristics studied and the available data was considerably less than the healthy group. Overground walking environment typically showed a higher influence of BWU than treadmill walking. We observed that kinetic gait parameters were more influenced by BWU than the kinematic ones but there is no consensus in literature for some of these parameters. However, upto 30% unloading, the influence of BWU on kinematic gait parameters seemed to be limited. This finding has wider implications on the effectiveness of BWST, since a natural gait may be maintained below 30% unloading. The distinction and subsequent investigation of these gait characteristics may help to unearth pivotal compensatory mechanisms in gait and serve as a reference document for conducting future studies on the effects of body weight unloading on human gait.

## Additional file

Additional file 1 Chronological listing of the literature examined in this report, where Y: if the study used randomized trials and NA: nothing is mentioned explicitly about randomization of trials(NA). (XLSX 56.9 kb)

## Abbreviations

AP: Antero-posterior
BWS: Body weight support
BWU: Body weight unloading
BWST: Body weight supported training
BF: Biceps femoris long head
BWSTT: Body weight supported treadmill training
COP: Centre of pressure
ECW: Energy cost of walking
EMG: Electromyography
GRF: Ground reaction forces
HR: Heart rate
iDLS: Initial double limb stance phase
LG: Lateral gastrocnemius
MG: Medial gastrocnemius
OH: Overground - control
ON: Overground - neuromuscular impairment
O-M: Overground modulated
O-UM: Overground unmodulated
ONN: Overground - neuromuscular impairment - non-paretic leg
ONP: Overground - neuromuscular impairment - paretic leg
RF: Rectus femoris ROM: Range of motion
SCI: Spinal cord injury
ST: Stance phase
SLS: Single limb stance phase
SW: Swing phase
TA: Tibialis anterior
TH: Treadmill - healthy
TN: Treadmill - neuromuscular impairment
TNN: Treadmill - neuromuscular impairment - non-paretic leg
TNP: Treadmill - neuromuscular impairment - paretic leg
T-M: Treadmill modulated
T-UM: Treadmill unmodulated
tDLS: Terminal double limb stance phase
