# Correction to: Influence of body weight unloading on human gait characteristics: a systematic review

**DOI:** 10.1186/s12984-018-0414-7

**Published:** 2018-08-08

**Authors:** Salil Apte, Michiel Plooij, Heike Vallery

**Affiliations:** 1 Mechanical, Maritime and Materials Engineering (3mE), TU Delft, Mekelweg 2, 2628 Delft, CD Netherlands; 2 Motekforce Link, Hogehilweg 18-C, 1101 Amsterdam, CD Netherlands; 3 Mechanical, Maritime and Materials Engineering (3mE), TU Delft, Mekelweg 2, 2628 Delft, CD Netherlands

## Correction

The original article [1] contained a major error whereby Fig. 1 mistakenly displayed a duplicate of Fig. 5. The correct version of Fig. 1 has now been restored and can be viewed ahead.

**Fig. 1:**
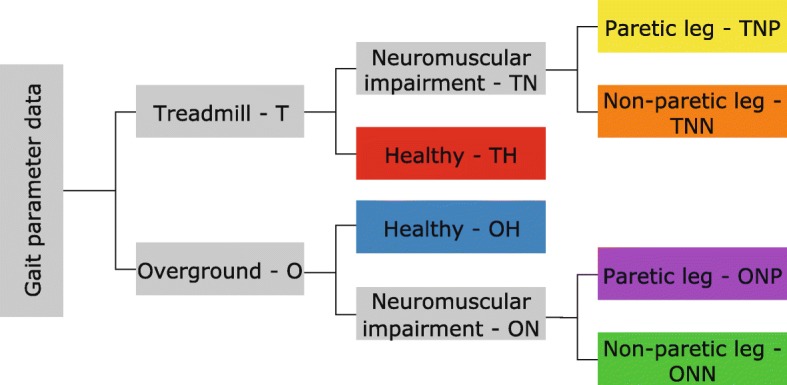
Flowchart for classification of studies into six categories which are indicated in colour. Similar colour scheme is followed in Figs. 3, 4, 5, and 6 in the results section

Furthermore, this error was mistakenly introduced by the production team that handled this article and as such, was not the fault of the authors.
